# Empowerment-based nutrition interventions on blood pressure: a randomized comparative effectiveness trial

**DOI:** 10.3389/fpubh.2023.1277355

**Published:** 2023-11-13

**Authors:** André Moreira-Rosário, Shámila Ismael, Inês Barreiros-Mota, Juliana Morais, Catarina Rodrigues, Inês Castela, Inês Curvelo Mendes, Maria Inês Soares, Luís Soares da Costa, Catarina Batista Oliveira, Tiago Henriques, Patrícia Pinto, Débora Pita, Catarina Marques de Oliveira, Janaína Maciel, Thaina Serafim, João Araújo, Júlio César Rocha, Diogo Pestana, Marta P. Silvestre, Cláudia Marques, Ana Faria, Jorge Polonia, Conceição Calhau

**Affiliations:** ^1^NOVA Medical School, Faculdade de Ciências Médicas, NMS, FCM, Universidade Nova de Lisboa, Lisbon, Portugal; ^2^CINTESIS@RISE, NOVA Medical School, Faculdade de Ciências Médicas, NMS, FCM, Universidade Nova de Lisboa, Lisbon, Portugal; ^3^CHRC, NOVA Medical School, Faculdade de Ciências Médicas, NMS, FCM, Universidade Nova de Lisboa, Lisbon, Portugal; ^4^CUF Academic and Research Medical Center, Lisbon, Portugal; ^5^Department of Community Medicine, Information and Health Decision Sciences (MEDCIDS), Faculty of Medicine, University of Porto, Porto, Portugal; ^6^CINTESIS@RISE, Faculty of Medicine, University of Porto, Porto, Portugal; ^7^Hypertension and Cardiovascular Risk Unit, Unidade Local de Saúde Matosinhos, Matosinhos, Portugal

**Keywords:** cardiovascular diseases, hypertension, blood pressure, mediterranean diet, sodium/potassium ratio

## Abstract

**Introduction:**

Empowerment lifestyle programs are needed to reduce the risk of hypertension. Our study compared the effectiveness of two empowerment-based approaches toward blood pressure (BP) reduction: salt reduction-specific program vs. healthy lifestyle general program.

**Methods:**

Three hundred and eleven adults (median age of 44 years, IQR 34–54 years) were randomly assigned to a salt reduction (*n* = 147) or a healthy lifestyle program (*n* = 164). The outcome measures were urinary sodium (Na^+^) and potassium (K^+^) excretion, systolic (SBP) and diastolic (DBP) blood pressure, weight, and waist circumference.

**Results:**

There were no significant differences in primary and secondary outcomes between the two program groups. When comparing each program to baseline, the program focused on salt reduction was effective in lowering BP following a 12-week intervention with a mean change of −2.5 mm Hg in SBP (95% CI, −4.1 to −0.8) and − 2.7 mm Hg in DBP (95% CI, −3.8 to −1.5) in the intention-to-treat (ITT) analysis. In the complete-case (CC) analysis, the mean change was −2.1 mm Hg in SBP (95% CI, −3.7 to −0.5) and − 2.3 mm Hg in DBP (95% CI, −3.4 to −1.1). This effect increases in subjects with high-normal BP or hypertension [SBP − 7.9 mm Hg (95% CI, −12.5 to −3.3); DBP − 7.3 mm Hg (95% CI, −10.2 to −4.4)]. The healthy lifestyle group also exhibited BP improvements after 12 weeks; however, the changes were less pronounced compared to the salt reduction group and were observed only for DBP [mean change of −1.5 mm Hg (95% CI, −2.6 to −0.4) in ITT analysis and − 1.4 mm Hg (95% CI, −2.4 to −0.3) in CC analysis, relative to baseline]. Overall, improvements in Na^+^/K^+^ ratio, weight, and Mediterranean diet adherence resulted in clinically significant SBP decreases. Importantly, BP reduction is attributed to improved dietary quality, rather than being solely linked to changes in the Na^+^/K^+^ ratio.

**Conclusion:**

Salt-focused programs are effective public health tools mainly in managing individuals at high risk of hypertension. Nevertheless, in general, empowerment-based approaches are important strategies for lowering BP, by promoting health literacy that culminates in adherence to the Mediterranean diet and weight reduction.

## 1. Introduction

Changing unhealthy lifestyle behaviors can decrease the prevalence of individuals with high blood pressure (BP) and cardiovascular diseases (CVD), contributing greatly to the sustainability of healthcare systems worldwide (1, 2). Current guidelines for managing hypertension recommend the adoption of a healthy diet as an integral part of disease treatment, regardless of antihypertensive medication intake (2–4).

Several dietary approaches have been proposed to reduce BP, including the Dietary Approaches to Stop Hypertension (DASH), the low-salt diet, and the Mediterranean diet (5–9). Recent systematic reviews of randomized controlled trials (RCTs) showed that dietary approaches with low sodium (Na^+^) and high potassium (K^+^) intake, such as DASH and low-salt diets, are effective in lowering BP (6, 7, 10). DASH and low-salt diets promote the consumption of nutrients and food components with antihypertensive properties such as minerals (potassium, magnesium, and calcium), vitamins, phytochemicals, polyphenols, unsaturated fatty acids, and fiber (11). Otherwise, the Mediterranean diet places greater emphasis on food groups and meals, rather than isolated nutrients. It is characterized by its elevated consumption of plant-based foods, such as fruits, vegetables, legumes, and nuts, while relying on olive oil as the main fat source. The diet also includes a moderate intake of fish and poultry and a reduced intake of dairy products, red and processed meats, and whole-fat dairy products (12). The protective effect of the Mediterranean diet against CVD has also been extensively studied (13). When compared to the DASH diet, the Mediterranean diet has demonstrated greater effectiveness in reducing the risk of CVD, particularly within populations already accustomed to these dietary and lifestyle practices (14).

However, in the context of preventing and treating hypertension, dietary interventions are mostly assessed individually. Yet, recent systematic reviews exploring both the DASH and Mediterranean diets have revealed that the DASH diet shows the most convincing proof of its efficacy in lowering BP (7, 8). Significantly, despite these insights, there has been no randomized trial so far that directly compares how the DASH and Mediterranean diets differ in their effects on reducing BP.

Furthermore, some of these studies use controlled and specific feeding methods to make sure participants stick to the planned diets. This is verified through close monitoring of participants during on-site meals, along with inquiries about their consumption of study foods, and the collection of urine samples. Although it is important to acknowledge that while these controlled scenarios play a critical role in assessing efficacy, they may not precisely mirror how these findings would apply to the daily circumstances of the broader population.

In the realm of encouraging changes in behavior, empowerment-based methods have emerged as powerful triggers, giving citizens the freedom to steer their own choices toward healthier eating preferences (15–19). However, despite its clear importance in improving health and well-being, the concept of empowerment has unfortunately not been used enough in programs designed to promote healthy dietary habits (15, 16). According to the World Health Organization (WHO), health promotion is a process that empowers individuals to have more control over the decisions and actions that affect their well-being (20). This broad and multifaceted view of health encompasses social, economic, and environmental factors, all of which play crucial roles in shaping daily health conditions (20, 21).

Hence, the driver to improve public health centers on spreading health information grounded in evidence, raising awareness, and empowering people to integrate personalized and suitable health-conscious behaviors into their daily routines.

With this viewpoint in mind, we designed a randomized comparative trial to thoroughly examine the effectiveness of two empowerment-driven approaches. One of these strategies focused on reducing salt intake, echoing the principles of the DASH diet, while the other centered around fostering a holistic and all-encompassing healthy lifestyle regimen. This latter approach incorporated guidance aligned with the core principles of the Mediterranean dietary pattern.

These educational efforts were carefully designed to enhance participants’ understanding of beneficial lifestyle practices. Moreover, the programs provided participants with practical resources to help them adopt new habits. This comprehensive toolkit encompassed strategies for embracing wholesome cooking practices and making well-informed choices when buying food. Essentially, our main goal was to determine which of these empowerment-focused methods would prove to be the more effective driver in nurturing health-oriented dietary habits and achieving reductions in BP across the broader population.

## 2. Materials and methods

### 2.1. Study design

This study is a multicenter, randomized, comparative effectiveness trial comparing the outcomes of two different 12-week empowerment-based approaches to promote healthy habits in the general population. The trial was conducted between March 2019 (the first candidate screened for eligibility) and September 2019 (the end of the 12-week follow-up of the last participant), after obtaining approval from the Ethical Committee of the Hospital CUF on December 18, 2018, for the project. The study was conducted in accordance with the ethical principles of the Declaration of Helsinki and followed the Good Clinical Practice guidelines. All enrolled participants provided voluntary, written informed consent. The present study adhered to the CONSORT reporting guidelines (Supplementary Table S1) and was registered on the ClinicalTrials.gov database (NCT03830021).

### 2.2. Participants

The study enrolled adult participants aged 20 to 70, who were responsible for acquiring and preparing their own meals, normal or with hypertension. Medicated hypertense individuals were included if medication and diet was stable for at least 3 weeks before the study. Eligible participants had to be willing and able to comply with the study protocol and provide informed consent. Exclusion criteria included a history of cardiovascular disease (such as ischemic cardiovascular disease, stable or unstable angina, myocardial infarction, stroke, or symptomatic peripheral arteriosclerosis), liver or kidney diseases, or cancer. Participants were also excluded if they were pregnant or breastfeeding women, women planning to become pregnant within the study period, had a history of drug, alcohol, or other substance abuse, or had other factors that might limit their ability to cooperate during the study.

### 2.3. Recruitment

Participants were recruited from the Lisbon Metropolitan Area through public advertisements in online newspapers and social media. Participants underwent eligibility screening and assessment at the study centers, which included the Hospital CUF Descobertas and Hospital CUF Infante Santo. Eligible participants were randomly assigned to one of two intervention groups (in a 1:1 ratio) using a computer-generated allocation sequence. The allocation was concealed through sequentially numbered, opaque, sealed envelopes. The allocation sequence was generated by a statistician who was not involved in recruitment or intervention delivery, ensuring that the allocation process was objective and unbiased. To maintain participant masking, the interventions were administered on different schedules, and participants were kept unaware of their assigned interventions.

### 2.4. Interventions

#### 2.4.1. Salt-reduction program

Participants randomized to the salt reduction program received a multi-component educational program for 12 weeks, consisting of three educational sessions that occurred during clinic visits (baseline, 4-week, and 8-week), five individual practical training sessions at the local supermarket, and 8 telephone counseling calls. During the initial educational session, participants received information regarding salt consumption, the health implications of excessive salt intake, and the foods they should avoid to reduce their salt intake. In the subsequent session, participants were educated on how to decipher food labels, make choices that have lower salt content within the same food group, and understand the significance of substituting salt with herbs and spices. In the final session, participants were enlightened about interpreting salt-related nutritional claims and the importance of meeting recommended fruit and vegetable intake. Additionally, the impact of potassium, calcium, and magnesium on BP was discussed, along with the identification of optimal dietary sources for these minerals. Following each session, participants were provided with an informational flyer covering the discussed topics. Participants in the salt reduction group benefitted from practical educational sessions conducted within supermarkets, facilitating the application of the acquired knowledge during the purchasing process. To further solidify the information transmitted during in-person education, telephone counseling calls were implemented between sessions.

#### 2.4.2. Healthy lifestyle program

Participants randomized to the healthy lifestyle program received a 12-week educational program that consisted of three sessions during clinic visits (at baseline, 4-weeks, and 8-weeks) and 12 telephone counseling calls. The first session focused on the impact of the Mediterranean diet on health, with an emphasis on cardiovascular health. It included recommendations on the best food choices and foods to avoid as part of the principles of the Mediterranean food pattern. The second session addressed various lifestyle topics, such as the importance of hydration, how to increase water intake, physical activity, and sleep quality. The third session focused on the negative health impact of addictive habits, such as alcohol consumption and smoking, as well as healthy culinary methods. After each session, participants received a flyer summarizing the topics discussed. Furthermore, they received four telephone counseling calls after each face-to-face session to reinforce the information and clarify any questions or doubts.

### 2.5. Outcome assessment

**Figure 1:**
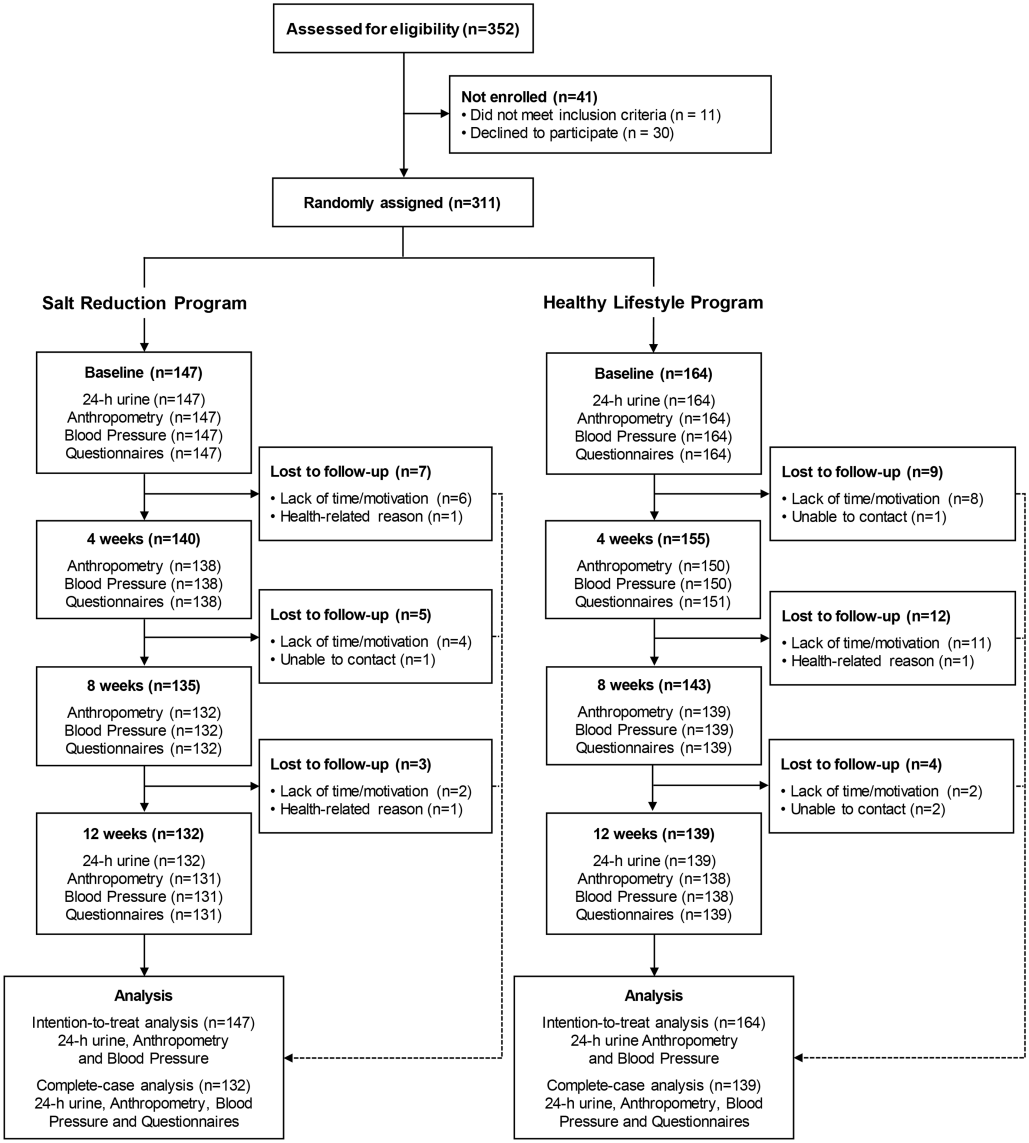
Flow diagram portraying the study design.

We collected 24-h urine samples at baseline and after the 12-week intervention period to estimate Na^+^ and K^+^ excretion. Secondary outcome measures including office BP, anthropometric measurements, and additional covariates (namely adherence to the Mediterranean diet), were measured at baseline and during follow-up at 4, 8, and 12 weeks (Figure 1). Participants were instructed on how to collect 24-h urine samples. Na^+^ and K^+^ in the urine were measured using flame photometry, and creatinine was measured using an automated validated enzymatic method at an authorized Clinical Analysis Laboratory (Centro de Medicina Laboratorial Germano de Sousa). We assessed the adequacy of collection based on the expected normal range of creatinine excretion, as previously described by Brenner and Rector (22). Since a large proportion of urinary samples fell outside the expected creatinine ranges, indicating inadequate urine collections, we used Tanaka formulas to estimate 24-h urinary Na^+^ and K^+^ excretion (23). We estimated salt intake from 24-h urinary sodium excretion as 1 mEq/24 h Na^+^ = 0.058 g per day salt. Office BP measurements were performed according to the guidelines of the European Society of Hypertension/European Society of Cardiology (24), using Omron M7 (HEM-780-E) oscillometric automated BP monitoring devices. These devices were purposefully acquired for their first use in the trial. These devices have been rigorously validated and achieved an ‘A/A’ performance classification under the British Hypertension Society (BHS) and Association for the Advancement of Medical Instrumentation (AAMI) SP10 requirements (25). Anthropometric measurements were performed according to the Directorate-General for Portuguese Health protocol for body weight, height, and waist circumference (26). Trained nutritionists, following a standardized protocol and strict quality control procedures, conducted both the BP and anthropometric measurements, including body weight, height, and waist circumference, during face-to-face clinic visits at Hospital CUF Descobertas and Hospital CUF Infante Santo. Within our Standard Operating Procedures (SOP), we diligently considered the following key factors regarding BP measurements: 1. Controlled the temperature of the clinical cabinet, maintaining it between 18 and 22°C; 2. Instructed and controlled the participants, ensuring they refrained from smoking or consuming stimulants such as coffee at least 60 min before the visit; 3. Allowed the participants to rest briefly before BP measurement; 4. Ensured that the participants positioned their supported measurement arm horizontally at the height of the heart during measurements; 5. Instructed the participants to place the sleeve of their shirt folded between the shoulder and the elbow, without exerting pressure on the arm and keeping their legs slightly open; 6. Enforced silence during BP measurements; 7. Conducted BP measurements in both arms during the recruitment visit, noting the arm with consistently higher pressure for subsequent visits; 8. At each appointment, BP was measured twice. If a significant discrepancy was observed between the initial measurements, a third measurement was taken into consideration. The questionnaire was used to collect relevant covariates, including socio-demographic and health information, dietary assessment, medication, smoking status, and protocol compliance. Adherence to the Mediterranean diet was evaluated using a previously validated 14-item questionnaire, known as the PREDIMED Mediterranean Diet Adherence Screener (MEDAS) (27). The MEDAS score was categorized as having the lowest adherence (score 0–5), average adherence (score 6–9), and highest adherence (score ≥ 10), as is commonly reported in the literature (28–30). The assessment of salt content in food purchased was not performed, as planned, due to a delay in the authorization from the grocery company.

### 2.6. Statistical analysis

The sample size calculation was based on the estimated difference in salt reduction between the two groups after 12 weeks. Assuming a salt reduction of 1 g/day and a standard deviation of 3.8 g/day (22), a sample size of 500 participants (250 per group) was calculated to provide 80% power at a 5% level of significance (two-sided) while taking into account a 10% dropout rate.

Descriptive statistics were reported as numbers and percentages for categorical variables and as mean and standard deviation for continuous variables, or median and interquartile ranges if the variable’s distribution was skewed. Between-group differences at baseline were assessed using appropriate tests such as the independent sample t-test, Mann–Whitney U test, or chi-squared test.

The effect of the intervention on changes in BP, 24-h urinary excretion of Na^+^ and K^+^, and anthropometry measurements such as weight, BMI, and waist circumference were assessed using ANCOVA with treatment group as the predictor and study center, age, sex, baseline antihypertensive medications, baseline systolic BP value, participant program protocol compliance, and smoking status as covariates. Protocol compliance was evaluated based on adherence to the sessions aimed to improve health literacy, as our primary goal was to empower participants and then, assess the effectiveness of two programs for salt reduction. To quantify participants’ adherence to the study protocol, we established a scoring system, and the score value was taken into consideration in the ANCOVA model. To manage missing data, multiple imputation was performed for intention-to-treat (ITT) analysis, using the chained equations approach with 5 imputed datasets and 10 iterations, and the results were pooled using Rubin’s rule (31). Sensitivity analyses were carried out to assess the robustness of the multiple imputation method by comparing the distribution plots of recorded values with imputed values. All analyses were conducted using SPSS version 27 software (SPSS Inc., Chicago, IL, United States).

## 3. Results

### 3.1. Recruitment and baseline characteristics of the participants

From March 29 to June 4, 2019, a total of 352 candidates were screened for eligibility. After an initial assessment, 30 declined to participate and 11 did not meet the inclusion criteria. Thus, 311 participants were enrolled, comprising 224 women and 87 men with a median age of 44 years (IQR 34–54 years), who were randomly assigned to either a salt reduction-focused program (*n* = 147) or a healthy lifestyle program (*n* = 164; see Figure 1).

The study ended before the estimated 500 participants were recruited due to lower than anticipated recruitment rates. All participants contributed to baseline data and their characteristics are shown in Table 1, according to the intervention group. The proportion of women was higher in the healthy lifestyle group (78.0% vs. 65.3%, *p* = 0.012), but demographic and clinical characteristics were otherwise balanced across the groups. The healthy lifestyle group had a slightly higher proportion of participants who withdrew from the study (15.2% vs. 10.2%, *p* = 0.185); the lack of time or motivation was the most frequent reason for discontinuation in both groups. None of the participants reported adverse effects.

**Table 1:** Baseline demographic and clinical characteristics of participants by randomized group.

Characteristic	Salt reduction program (*n* = 147)	Healthy lifestyle program (*n* = 164)	*P v*alue
Age, y	44 [34–52]	44 [35–55]	0.688
Sex, female	96 (65.3%)	128 (78.0%)	**0.012**
Ethnicity			
White/European	137 (93.2%)	151 (92.1%)	0.842
White/African or South American	6 (4.1%)	7 (4.3%)	
Black	1 (0.7%)	3 (1.8%)	
Mixed	3 (2.0%)	3 (1.8%)	
Weight, kg	75.6 (14.3)	74.5 (18.2)	0.570
BMI, kg/m^2^	27.1 (4.7)	27.6 (6.0)	0.430
Overweight/Obese (≥25 BMI)	92 (62.6%)	99 (60.4)	0.688
Waist circumference, cm	85.7 (11.9)	85.0 (15.5)	0.649
Smoking status			
Current smoker	19 (12.9%)	23 (14.0%)	0.761
Former smoker	29 (19.7%)	37 (22.6%)	
Married or cohabiting	126 (85.7%)	132 (80.5%)	0.221
Professionally active	124 (84.4%)	128 (78.0%)	0.157
Education			
University	111 (75.5%)	122 (74.4%)	0.820
Secondary or lower	36 (24.5%)	42 (25.6%)	
Self-reported medical disorders			
Hypertension	25 (17.0%)	28 (17.1%)	0.988
Diabetes	4 (2.7%)	2 (1.2%)	0.336
Dyslipidemia	39 (26.5%)	42 (25.6%)	0.853
Hypothyroidism	3 (2.0%)	10 (6.1%)	0.074
Hyperthyroidism	1 (0.7%)	4 (2.4%)	0.218
Family history of hypertension, dyslipidemia, or CVD	99 (67.3%)	109 (66.5%)	0.869
MEDAS, score			
Low adherence (score ≤ 5)	26 (17.7%)	24 (14.6%)	0.511
Average adherence (score 6–9)	94 (63.9%)	115 (70.1%)	
High adherence (score ≥ 10)	27 (18.4%)	25 (15.2%)	
Office measurements			
Systolic blood pressure, mm Hg	116.1 (15.0)	115.4 (15.5)	0.670
Diastolic blood pressure, mm Hg	75.1 (9.9)	76.0 (9.1)	0.446
Heart rate, beats per minute	71.7 (11.0)	73.8 (10.2)	0.088
Urinary excretion (Tanaka prediction)			
Sodium, mmol/24 h	157.2 (26.3)	155.6 (23.8)	0.572
Potassium, mmol/24 h	48.6 (6.7)	47.9 (6.7)	0.384
Salt intake estimated, g/d	9.2 (1.5)	9.1 (1.4)	0.572
Antihypertensive medications	29 (19.7%)	41 (25.0%)	0.125

Data are number of participants (%), mean (standard deviation), and median (interquartile range). CVD, cardiovascular diseases; BMI, body mass index; BP, blood pressure. p values were calculated using independent samples t test, Mann–Whitney U test or chi-squared test as appropriate.

Most participants were white Europeans (92.6%), professionally active (81.0%), and had a university degree (74.9%). Additionally, over half were overweight or obese (61.4%) and had an average adherence to a Mediterranean diet (67.2%) at baseline. Two-thirds of the participants had a family history of hypertension, dyslipidemia, or CVD (66.9%), while almost one-fourth had dyslipidemia (26.0%). At study entry, 17.0% of participants reported having hypertension, and 22.5% were taking antihypertensive medication. At baseline, the mean systolic/diastolic BP was 116/76 (SD 15/10) mmHg, and the estimated mean 24-h urinary Na^+^ excretion was 156.3 (SD 24.9) mmol/day.

The study’s primary and secondary outcome measures are presented in Table 2. To evaluate the effectiveness of the interventions, both intention-to-treat (ITT) and complete-case (CC) analyses were conducted. The ITT analysis included all participants who were randomized and is considered the most reliable method of analysis, while the CC analysis only included participants who completed the study and may overestimate the intervention’s effectiveness. By presenting results from both the ITT and CC analyses, we provide a more comprehensive understanding of the intervention’s effectiveness, accounting for both ideal and real-world scenarios. This approach ensures that the study’s findings are robust and applicable to clinical practice.

**Table 2:** Mean difference in outcome measures after 12 weeks.

	Complete-case					Intention-to-treat				
	Salt reduction program (*n* = 132)		Healthy lifestyle program (*n* = 139)		*P* value between groups	Salt reduction program (*n* = 147)		Healthy lifestyle program (*n* = 164)		*P* value between groups
	*n*	Mean change from baseline (95% CI)	*n*	Mean change from baseline (95% CI)		*n*	Mean change from baseline (95% CI)	*n*	Mean change from baseline (95% CI)	
Office measurements										
Systolic blood pressure, mm Hg	131	−2.5 (−4.1, −0.8)*	138	−1.1 (−2.8, 0.5)	0.263	147	−2.1 (−3.7, −0.5)*	164	−0.7 (−2.3, 0.9)	0.237
Diastolic blood pressure, mm Hg	131	−2.7 (−3.8, −1.5)*	138	−1.4 (−2.4, −0.3)*	0.107	147	−2.3 (−3.4, −1.1)*	164	−1.5 (−2.6, −0.4)*	0.330
Heart rate, beats per minute	131	−2.1 (−3.7, −0.6)*	138	−1.6 (−3.1, −0.1)*	0.655	147	−1.8 (−3.3, −0.3)*	164	−1.6 (−3.1, −0.2)*	0.873
Urinary excretion (Tanaka prediction)										
Sodium, mmol/24 h	132	0.0 (−4.8, 4.8)	139	−1.1 (−5.8, 3.6)	0.754	147	0.1 (−4.4, 4.6)	164	−0.4 (−4.6, 3.9)	0.880
Potassium, mmol/24 h	132	1.1 (−0.3, 2.4)	139	1.9 (0.6, 3.2)*	0.413	147	1.4 (0.1, 2.6)*	164	2.1 (0.9, 3.3)*	0.400
Salt intake estimated, g/d	132	0.0 (−0.3, 0.3)	139	−0.1 (−0.3, 0.2)	0.754	147	0.0 (−0.3, 0.3)	164	0.0 (−0.3, 0.2)	0.880
Sodium/potassium ratio	132	0.0 (−0.1, 0.1)	139	−0.1 (−0.2, 0.0)	0.321	147	0.0 (−0.1, 0.0)	164	−0.1 (−0.2, 0.0)	0.719
Weight, kg	131	−0.3 (−0.7, 0.0)	138	−0.3 (−0.7, 0.0)	0.965	147	−0.5 (−1.5, 0.4)	164	−0.3 (−1.2, 0.7)	0.698
BMI, kg/m^2^	131	−0.1 (−0.2, 0.1)	138	−0.1 (−0.2, 0.1)	0.813	147	−0.2 (−0.6, 0.2)	164	−0.1 (−0.5, 0.3)	0.831
Waist circumference, cm	131	0.0 (−0.7, 0.7)	135	−0.3 (−1.0, 0.4)	0.539	147	−0.1 (−1.3, 1.0)	164	0.2 (−0.9, 1.3)	0.696

Values are mean (95% CI). ANCOVA models were adjusted for study center, age, sex, antihypertensive medications, baseline systolic blood pressure value, participant program protocol compliance and smoking status. *p value is statistically significant.

After the 12-week intervention, there were no significant differences observed between the salt reduction-focused and healthy lifestyle programs regarding predicted 24-h urinary Na^+^ and K^+^ excretion, as well as systolic and diastolic blood pressure (SBP and DBP), weight, and waist circumference. Nonetheless, the salt reduction program led to noteworthy enhancements in BP compared to baseline, with a mean change of −2.5 mm Hg in SBP (95% CI, −4.1 to −0.8) and − 2.7 mm Hg in DBP (95% CI, −3.8 to −1.5) in the intention-to-treat (ITT) analysis, while −2.1 mm Hg in SBP (95% CI, −3.7 to −0.5) and − 2.3 mm Hg in DBP (95% CI, −3.4 to −1.1) in the complete-case (CC) analysis. Notably, the healthy lifestyle group also exhibited BP improvements after 12 weeks; however, these were less pronounced compared to the salt reduction group and were observed only for DBP [mean change of −1.5 mm Hg (95% CI, −2.6 to −0.4) in ITT analysis and − 1.4 mm Hg (95% CI, −2.4 to −0.3) in CC analysis, relative to baseline].

The reduction in BP within the groups may be attributed to the enhancement in predicted 24-h K^+^ excretion after the 12-week intervention compared to baseline. This positive trend was observed in both groups during the ITT, with a mean change of 2.1 mmol/24 h (95% CI, 0.9 to 3.3) for the healthy lifestyle group and 1.4 mmol/24 h (95% CI, 0.1 to −2.6) for the salt reduction group. In the CC analysis, this improvement was relatively smaller, achieving statistical significance solely within the healthy lifestyle group at 1.9 mmol/24 h (95% CI, 0.6 to 3.2).

### 3.2. Impact of intervention on blood pressure

**Figure 2:**
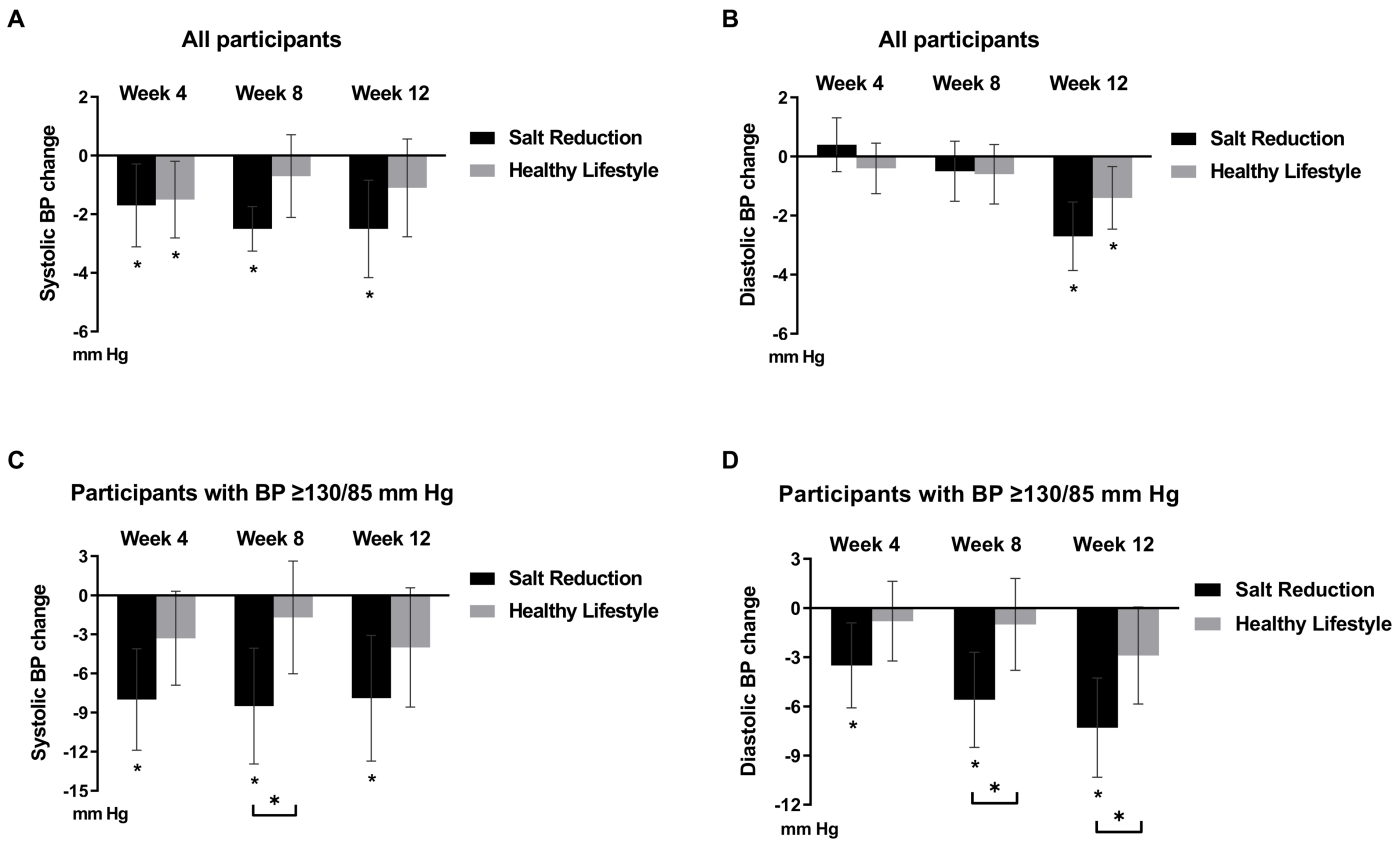
Change in office blood pressure (BP) from baseline to 4, 8 and 12 weeks in the complete-case population. All participants from salt reduction and healthy lifestyle groups were included in the analysis of systolic BP **(A)** and diastolic BP **(B)**. Subgroup analysis of participants with high-normal or hypertension at baseline (SBP ≥ 130 and/or DBP ≥ 85, mm Hg; *n* = 32 in the healthy lifestyle group and *n* = 30 in the salt reduction group) were also included in the analysis of systolic BP **(C)** and diastolic BP **(D)**. Data are presented as mean (95% CI), adjusted for study center, age, sex, antihypertensive medications, baseline systolic blood pressure value, participant program protocol compliance and smoking status (ANCOVA). *p value is statistically significant.

After 4 weeks, both the salt reduction and healthy lifestyle programs led to lower SBP compared to baseline: −1.7 mm Hg (95% CI, −3.1 to −0.3) for the salt reduction and − 1.5 mm Hg (95% CI, −2.8 to −0.2) for the healthy lifestyle. Importantly, the salt reduction group maintained lower SBP at 8 and 12 weeks, unlike the healthy lifestyle group (Figure 2A). Both groups showed DBP improvement after 12 weeks (Figure 2B). Moreover, there were no significant sex differences in BP outcomes (data not shown).

In participants with high-normal or hypertension (SBP ≥ 130 and/or DBP ≥ 85, mm Hg; *n* = 32 in the healthy lifestyle group and *n* = 30 in the salt reduction group), a subgroup analysis revealed significant BP reduction after 12 weeks within the salt reduction group: SBP decreased by −7.9 mm Hg (95% CI, −12.5 to −3.3), and DBP decreased by −7.3 mm Hg (95% CI, −10.2 to −4.4). Furthermore, notable differences between the groups were observed at week 8 in both SBP [−6.8 mm Hg (95% CI, −12.8 to −0.7), *p* = 0.029] and DBP [−4.6 mm Hg (95% CI, −8.6 to −0.6), *p* = 0.025]. Interestingly, a significant between-group difference in DBP was also evident at the end of the 12-week intervention [−4.4 mm Hg (95% CI, −8.7 to −0.2), *p* = 0.041], favoring the salt reduction program (Figures 2C,D).

### 3.3. Impact of Na^+^/K^+^ ratio on blood pressure

**Figure 3:**
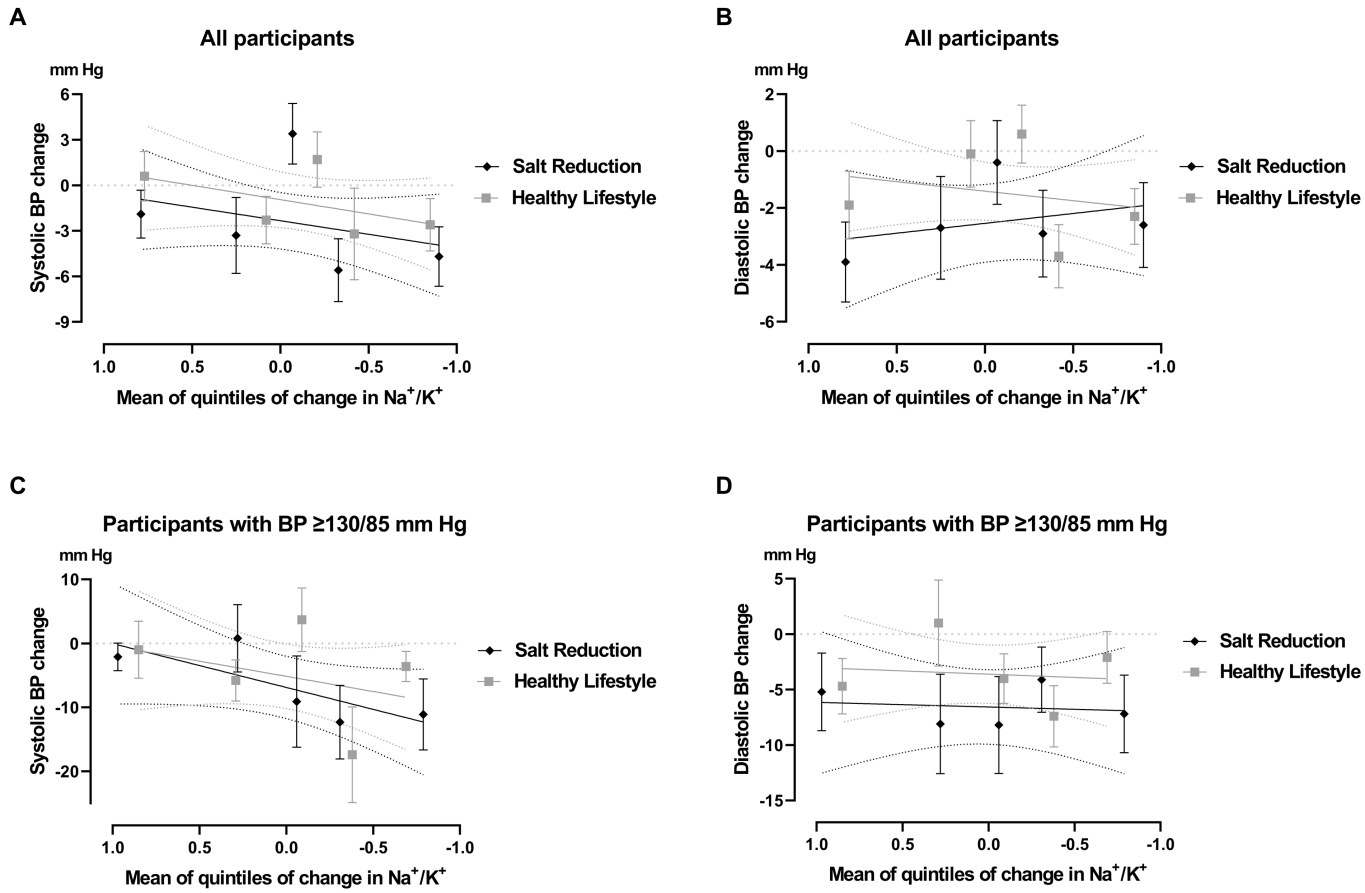
Change in office blood pressure (BP) stratified by mean quintiles of changes in Na^+^/K^+^ ratio, after 12-week follow-up. Complete-case participants from salt reduction and healthy lifestyle groups were included in the analysis of systolic BP **(A)** and diastolic BP **(B)**. Subgroup analysis of participants with high-normal or hypertension at baseline (SBP ≥ 130 and/or DBP ≥ 85, mm Hg) were also included in the analysis of systolic BP **(C)** and diastolic BP **(D)**. Data are presented as mean (SEM). Significance between Q1 to Q5 quintile subgroups was assessed by one-way analysis of variance (ANOVA) corrected with Bonferroni test for multiple comparisons. Differences in the same quintile between salt reduction and healthy lifestyle groups were assessed by independent t test.

Participants were categorized into quintile groups (Q1 to Q5) based on changes in the 12-week Na^+^/K^+^ ratio relative to the baseline for each program. The quintile groups represent the range of changes from lowest to highest. The variations in BP across these quintile groups are shown in Figure 3.

While not reaching statistical significance, these findings suggest that a lower Na^+^/K^+^ ratio tends to correspond with a reduction in mean SBP variation. This reduction ranges from −1.9 mm Hg (95% CI, −5.1 to 1.4) to −4.8 mm Hg (95% CI, −8.7 to −0.6) for the salt reduction program, and from 0.6 mm Hg (95% CI, −2.8 to 3.9) to −2.6 mm Hg (95% CI, −6.2 to 0.9) for the healthy lifestyle program, when comparing the lowest (Q1) to the highest (Q5) quintiles. This observed tendency remains consistent even when considering participants with high-normal or hypertension at baseline, as illustrated in Figure 3C. Notably, within this subgroup, the slope is more pronounced in the salt reduction group, ranging from −2.1 mm Hg (95% CI, −7.7 to 3.4) to −11.1 mm Hg (95% CI, −25.3 to 3.1), whereas the healthy lifestyle group displays a modest decrease from −1.0 mm Hg (95% CI, −12.0 to 9.9) to −3.6 mm Hg (95% CI, −9.7 to 2.6).

### 3.4. Impact of weight and adherence to the mediterranean diet on blood pressure

**Figure 4:**
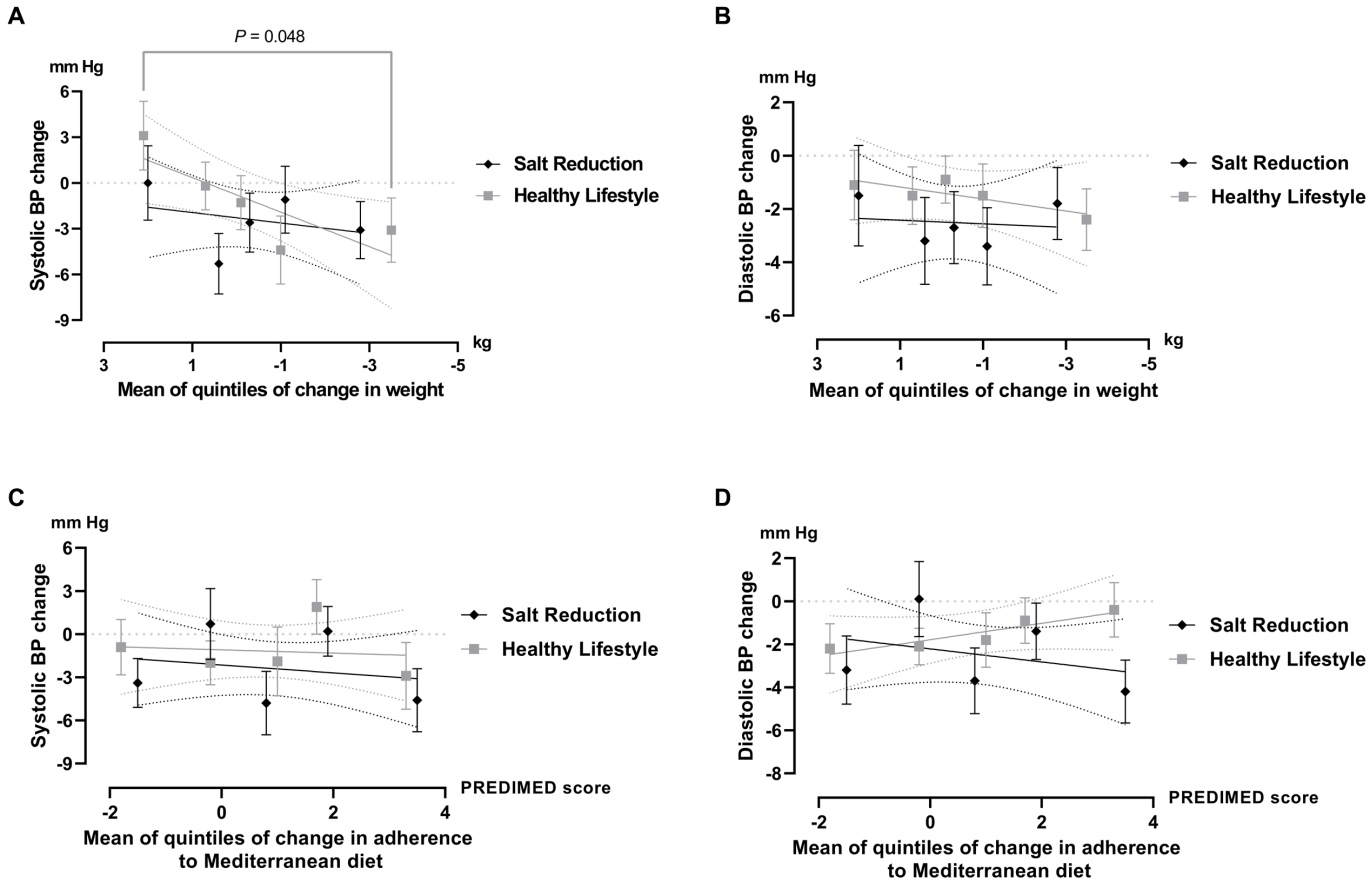
Change in office blood pressure (BP) stratified by mean quintiles of changes in weight **(A,B)** and adherence to Mediterranean diet **(C,D)**, after 12-week follow-up. Complete-case participants from salt reduction and healthy lifestyle groups were included in the analysis of systolic BP and diastolic BP. Data are presented as mean (SEM). Significance between Q1 to Q5 quintile subgroups was assessed by one-way analysis of variance (ANOVA) corrected with Bonferroni test for multiple comparisons. Differences in the same quintile between salt reduction and healthy lifestyle groups were assessed by independent t test.

For each program, participants were divided into Q1 to Q5 quintile groups based on weight changes after 12 weeks compared to baseline (lowest to highest change). We then analyzed how changes in SBP and DBP related to these weight quintile groups (shown in Figures 4A,B). Results indicate that higher SBP reductions are linked with greater weight loss, particularly in the higher weight reduction quintiles (Figure 4A). Notably, participants in the highest weight change quintile (Q5) experienced a − 3.1 mm Hg reduction in SBP for both programs. However, this trend is more pronounced in the healthy lifestyle group (Figure 4A). Importantly, a significant difference in SBP is observed in the healthy lifestyle group when comparing Q1 and Q5 quintile groups [6.2 mm Hg (95% CI, 0.0 to 12.4), *p* = 0.048].

Adherence to the Mediterranean diet was assessed using a well-established 14-item questionnaire known as the Mediterranean Diet Adherence Screener (MEDAS). Based on MEDAS scores, there was a noteworthy increase in mean adherence to the Mediterranean diet within both groups after the 12-week period: 0.9 (0.6 to 1.2, *p* = 0.001) for the salt reduction program and 0.8 (0.5 to 1.1, *p* = 0.001) for the healthy lifestyle program (data not shown). These changes in score seemed to be driven by increased intake of specific dietary components, namely vegetables, fruits, fish or seafood, tree nuts, and dishes seasoned with sofrito (sofrito is a seasoning blend commonly used in Mediterranean cuisine, made with chopped onions, garlic, and other aromatic ingredients sautéed with olive oil). Simultaneously, there was a decrease in the consumption of red or processed meats, butter, cream, margarine, soda drinks, sweets, and confectionery (Supplementary Figure S1). Moreover, no differences between-group were observed in terms of the total MEDAS score or between specific dietary components (data not shown).

Subsequently, participants were categorized into quintile groups based on the extent of their changes in adherence to the Mediterranean diet following the 12-week follow-up in comparison to their baseline measurements (Figures 4C,D); the stratification was done for each intervention program. This analysis demonstrates that participants who exhibited the most significant shift toward adherence to the Mediterranean diet experienced a modest yet noticeable reduction in SBP within both programs.

Furthermore, an investigation was undertaken to explore the relationship between Na+/K+ ratios and enhanced adherence to the Mediterranean diet (Supplementary Table S2). As anticipated, individuals with the highest adherence to the Mediterranean diet (MEDAS score ≥ 10) exhibited a notably lower mean Na^+^/K^+^ ratio, in comparison to both the average MEDAS score (*p* = 0.031) and the lowest adherence group (*p* = 0.009, Supplementary Table S2). This comparison suggests that the reduction in the Na^+^/K^+^ ratio is associated with adherence to the intervention programs, which, in turn, corresponds with adhering to the Mediterranean diet.

## 4. Discussion

In the broader effort to address the impact of high BP and CVD across populations, there is a pressing need to establish effective strategies for encouraging behavior changes (1, 2). However, implementing these strategies is challenging for healthcare professionals. Thus, we conducted a randomized trial to assess the impact of two distinct empowerment-focused approaches on dietary habits and BP, as endorsed by clinical nutrition experts. Our goal was to identify key factors in lifestyle adjustments that contribute to successful BP reduction. To achieve this, we compared a salt reduction program with a holistic healthy lifestyle approach.

**Figure 5:**
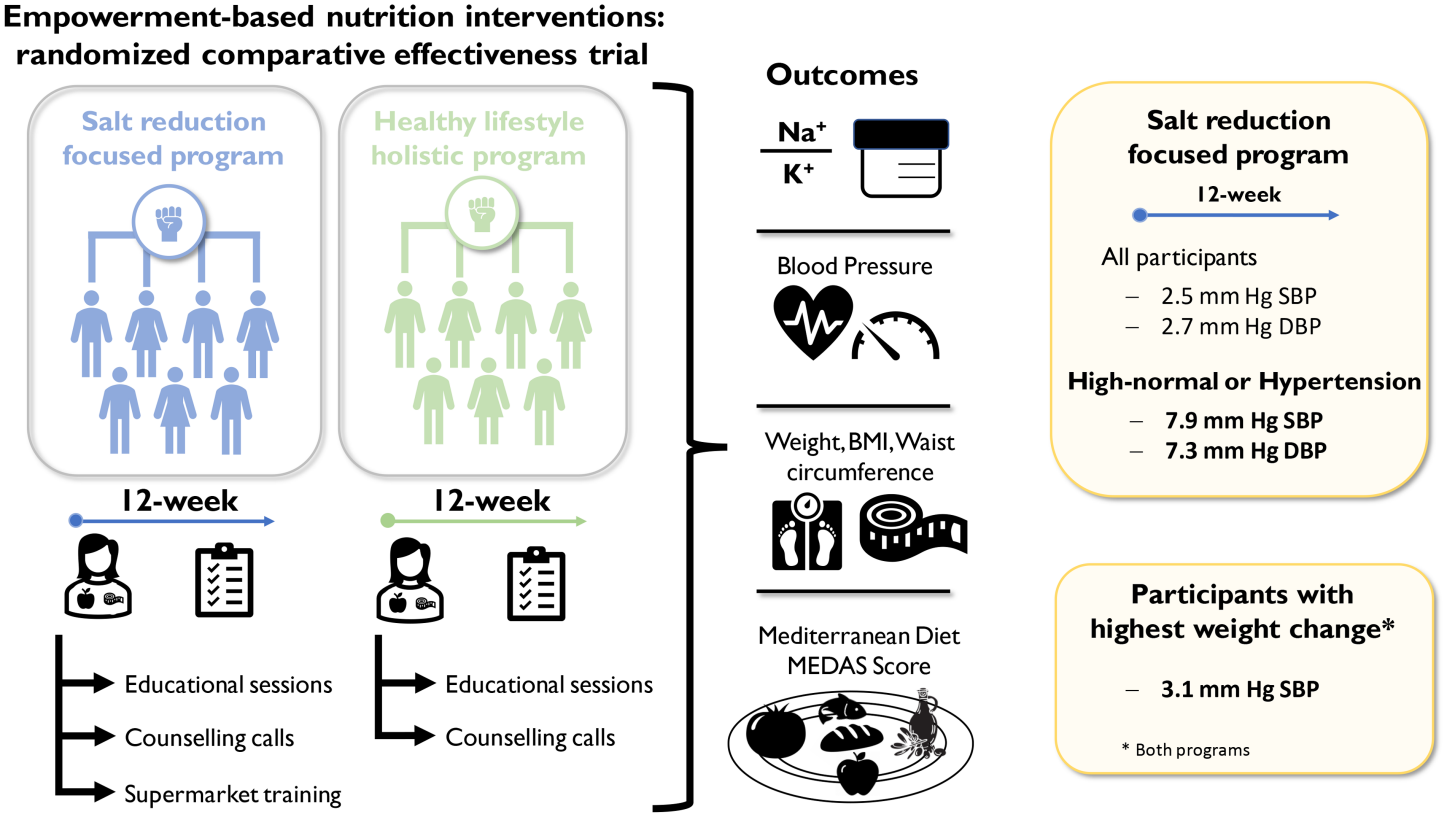
Schematic representation of the clinical trial design, showing the major characteristics of the intervention groups. The outcome variables are highlighted as well as the major findings.

Our findings revealed that both interventions were effective in reducing DBP after 12 weeks. However, only the intervention focused on salt reduction was significantly effective in decreasing SBP, with a substantial mean reduction of −2.5 mm Hg after 12 weeks compared to baseline. Furthermore, the salt reduction program was more effective at reducing BP in participants with high-normal or hypertension at baseline (SBP ≥ 130 and/or DBP ≥ 85, mm Hg; Figure 5). This highlights the notion that customizing recommendations to address specific public health concerns, such as hypertension, can lead to a more pronounced impact, especially within risk groups These results are in line with recent systematic reviews that emphasize the superior efficacy of both the DASH and low-salt diet in lowering BP when compared to the Mediterranean diet (6–8, 10). However, it is crucial to emphasize that while distinctions were noticeable, especially among participants with high-normal BP or hypertension, there were no statistically significant differences observed across all study participants.

The magnitude of our results in a short period is outstanding and consolidates the importance of diet and nutrition in the management of hypertension, independently of pharmacology. Because BP decrease is semilogarithmic associated with the incidence of cardiovascular outcomes, even a minor reduction has significant benefits (7). Undeniably, this improvement in BP is highly relevant since previous studies demonstrated that even a 2 mmHg reduction in SBP and DBP is associated with 10% lower stroke-related mortality and with a 7% lower risk of coronary artery disease (6). Likewise, 2 mmHg reduction in SBP substantially reduced the risk of CVD (27 events for coronary heart disease, 24 events for stroke, and 41 events for heart failure per 100,000 person-years) (32).

Nevertheless, it was somewhat surprising that the interventions yielded no significant impact on Na^+^ and K^+^ concentrations. It is noteworthy to emphasize that our study design involved participants attending dietary educational sessions, resembling intervention studies focused on the Mediterranean diet (8), as opposed to the controlled feeding protocols seen in DASH trials (7). This suggests that the reduced BP is more likely a result of an overall improvement in dietary quality rather than changes in the Na^+^/K^+^ ratio, as previously emphasized (9).

Indeed, the observed decrease in BP in our study can be attributed to the intentional behavior changes adopted by participants over the 12-week intervention period. Specifically, participants increased their consumption of vegetables, fruits, fish, tree nuts and dishes seasoned with sofrito, while reducing their intake of processed meats, butter, margarine, and high-sugar products. Significantly, these dietary changes comprise beneficial food ingredients that could account for the observed decrease in BP. Notably, the presence of vitamins and flavonoids in vegetables and fruits might induce blood vessel relaxation, driven by their antioxidant and anti-inflammatory properties (33). Similarly, fish consumption, attributed to its long-chain n-3 polyunsaturated fatty acids, is associated with a moderate reduction in BP, possibly by enhancing vascular reactivity and endothelial function (34). The diverse range of nutrients in nuts, including polyunsaturated fatty acids, magnesium, and antioxidants, could potentially confer a favorable impact on BP (35). Importantly, the olive oil within sofrito is rich in bioactive phenolic compounds that may enhance endothelial function by increasing nitric oxide availability and triggering vasodilation (36).

Furthermore, these dietary modifications yielded improvements in various anthropometric measures, ultimately contributing to the reduction in BP. While variations in body measurements did not result in a significant mean difference, a noticeable trend toward weight reduction was apparent. It is worth noting that weight loss constitutes a significant lifestyle factor in the prevention and management of hypertension, often influenced by dietary choices and physical activity (37). Importantly, our clinical trial indicates that participants who achieved more substantial weight reduction within the highest quintiles also observed greater reductions in BP. Interestingly, the slope of this trend was more pronounced in the healthy lifestyle group. Nevertheless, both groups reduced −3.1 mm Hg SBP in the highest quintiles of weight change. These findings corroborate a recent systematic review and meta-analysis (11), indicating that larger variations in body weight are associated with a more pronounced impact on BP. Similarly, a comprehensive dose–response meta-analysis (38) revealed that each 1 kg of weight loss corresponds to an approximate 1 mmHg reduction in SBP.

As expected, participants who achieved the largest reductions in Na^+^/K^+^ ratio exhibited a trend toward lower SBP, especially among individuals with high-normal or hypertension at baseline, although the results did not attain statistical significance. Importantly, following the 12-week intervention, participants with the highest adherence to the Mediterranean diet exhibited a significantly lower mean Na^+^/K^+^ ratio. This was expected since increased urinary K^+^ excretion is associated with a higher intake of vegetables and fruit, whole grains, low-fat dairy products, fish, and poultry, all endorsed in the Mediterranean diet. Likewise, lower excretion is associated with an unhealthy diet, including calorie-dense foods such as fast food and high-energy drinks. Furthermore, urinary K^+^ measurement is correlated with surrogate outcomes, such as heart rate and BP, and is a predictor of both all-cause and cause-specific mortality in the general population (39).

According to current hypertension prevention guidelines, lifestyle changes such as a healthy diet are recommended for all patients as they can delay or complement ongoing treatment (40). Our study provides evidence and guidance to support the adoption of behavioral approaches in clinical settings as effective strategies to promote healthy habits. These approaches include improving population education through health information, awareness, and knowledge. Therefore, the proposed interventions are crucial as they facilitate long-term healthy behavioral changes, improve health outcomes, and counteract the growing prevalence of unhealthy diets.

The strengths of our study include a randomized design and a notably ample sample size. We employed an interdisciplinary empowerment-based approach by collaborating with a multidisciplinary team of 12 nutritionists operating within clinical and grocery shopping settings. Furthermore, we adopted a pragmatic approach, involving participants with hypertension and those using antihypertensive medications. Through this deliberate inclusion of individuals from this high-risk group, the applicability of the study findings to a broader population is enhanced, thereby increasing the generalizability of our findings. Lastly, we followed a standardized protocol and strict quality control procedures for clinical measurements and data collection, thus ensuring the accuracy and consistency of our data.

On the other hand, this trial presents several limitations that warrant acknowledgment. Firstly, differences in group retention were noted, with a lower attrition rate observed among participants in the salt reduction program compared to the holistic healthy lifestyle group. This divergence could be attributed to the comparatively reduced contact inherent to the healthy lifestyle program, potentially leading to a diminished level of motivation among these participants. This variance in follow-up could potentially introduce bias favoring the salt reduction group. However, intention-to-treat analyses employing multiple imputation methods, to address missing data, yielded outcomes consistent with the complete-case analysis. Secondly, there was an imbalance in the sex distribution, with a higher proportion of women in the healthy lifestyle group. Nonetheless, we addressed this disparity by incorporating sex and participant compliance as covariates in the analysis to mitigate potential confounding effects. Thirdly, the study concluded before attaining the intended sample size of 500 participants. Although the study still encompassed a relatively substantial sample size, the failure to reach the target number might have impacted the statistical power to discern differences in outcome assessments between the groups. Fourth, due to ethical considerations, the study lacked a no-intervention control group, and the design did not allow double-blinding. Fifth, it is worth noting that participants who volunteer for dietary trials generally exhibit a higher degree of motivation to adhere to a dietary program compared to the broader population. Furthermore, most participants were professionally active and had higher levels of education, potentially facilitating a greater assimilation of knowledge. Consequently, the outcomes of the programs may not be as effective in the general population.

## 5. Conclusion

This study shows that empowerment-based approaches, aimed at promoting healthy culinary habits and improved purchasing options, effectively lower BP in the short term. However, an intervention focused on educating participants about salt reduction was found to be more impactful in lowering both systolic and diastolic BP, particularly in those with high-normal or hypertensive BP. Moreover, approaches that promote adherence to the Mediterranean diet were associated with weight loss and a decrease in the Na^+^/K^+^ ratio, resulting in improved BP. Importantly, the decrease in BP primarily results from an overall improvement in dietary quality, rather than being solely attributed to changes in the Na^+^/K^+^ ratio. These findings highlight the importance of targeted lifestyle interventions and the potential benefits of a Mediterranean-style diet in BP management. Thus, the study results highlight the importance of promoting healthy lifestyle practices through empowerment, aiming to prevent the onset of hypertension and ameliorate advanced stages of elevated BP. This approach could contribute to reducing the risk of potential complications. Therefore, it is imperative to define new strategies that mirror a similar reduction program promoted by registered nutritionists and dietitians, which can provide the tools to the population for making healthier choices. This can contribute to reducing long-term health costs and improving the quality of life for the general population.

## Data availability statement

The original contributions presented in the study are included in the article/Supplementary material, further inquiries can be directed to the corresponding author.

## Ethics statement

The studies involving humans were approved by Ethical Committee of the Hospital CUF. The studies were conducted in accordance with the local legislation and institutional requirements. The participants provided their written informed consent to participate in this study.

## Author contributions

AM-R: Conceptualization, Data curation, Formal analysis, Investigation, Methodology, Supervision, Validation, Visualization, Writing – original draft. SI: Data curation, Investigation, Writing – review & editing. IB-M: Data curation, Investigation, Writing – review & editing. JM: Data curation, Investigation, Writing – review & editing. CR: Data curation, Investigation, Writing – review & editing. IC: Data curation, Investigation, Writing – review & editing. IM: Data curation, Writing – review & editing. MIS: Data curation, Writing – review & editing. LC: Data curation, Writing – review & editing. CBO: Data curation, Writing – review & editing. TH: Data curation, Writing – review & editing. PP: Data curation, Writing – review & editing. DÉP: Data curation, Writing – review & editing. CMO: Data curation, Writing – review & editing. JM: Data curation, Writing – review & editing. TS: Data curation, Writing – review & editing. JA: Writing – review & editing. JR: Writing – review & editing. DIP: Writing – review & editing. MPS: Writing – review & editing. CM: Writing – review & editing. AF: Writing – review & editing. JP: Conceptualization, Formal analysis, Investigation, Supervision, Validation, Visualization, Writing – review & editing. CC: Conceptualization, Formal analysis, Funding acquisition, Investigation, Supervision, Validation, Visualization, Writing – review & editing.
